# Impacts of social isolation stress in safety learning and the structure of defensive behavior during a spatial-based learning task involving thermal threat

**DOI:** 10.3389/fnbeh.2024.1503097

**Published:** 2024-12-10

**Authors:** Stephanie A. Villalon, Ada C. Felix-Ortiz, Kelly Lozano-Ortiz, John R. McCarrey, Anthony Burgos-Robles

**Affiliations:** ^1^Department of Neuroscience, Developmental, and Regenerative Biology, University of Texas at San Antonio, San Antonio, TX, United States; ^2^Department of Cellular and Integrative Physiology, University of Texas Health Science Center, San Antonio, TX, United States; ^3^Institute of Regenerative Medicine, University of Texas at San Antonio, San Antonio, TX, United States; ^4^Brain Health Consortium, The University of Texas at San Antonio, San Antonio, TX, United States

**Keywords:** fear, anxiety, conflict, decision making, behavioral flexibility, prefrontal cortex (PFC)

## Abstract

Safety learning during threat and adversity is critical for behavioral adaptation, resiliency, and survival. Using a novel mouse paradigm involving thermal threat, we recently demonstrated that safety learning is highly susceptible to social isolation stress. Yet, our previous study primarily considered male mice and did not thoroughly scrutinize the relative impacts of stress on potentially distinct defensive mechanisms implemented by males and females during the thermal safety task. The present study assessed these issues while considering a variety of defensive behaviors related to safety-seeking, escape, coping, protection, ambivalence, and risk-taking. After a two-week social isolation stress period, mice were required to explore a box arena that had thermal threat and safety zones (5 vs. 30°C, respectively). Since visuospatial cues clearly differentiated the threat and safety zones, the majority of the no-stress controls (69–75%) in both sexes exhibited optimal memory formation for the safety zone. In contrast, the majority of the stress-exposed mice in both sexes (69–75%) exhibited robust impairment in memory formation for the safety zone. Furthermore, while the control groups exhibited many robust correlations among various defensive behaviors, the stress-exposed mice in both sexes exhibited disorganized behaviors. Thus, stress severely impaired the proper establishment of safety memory and the structure of defensive behavior, effects that primarily occurred in a sex-independent manner.

## Introduction

Safety learning shapes behavior during threat and adversity to prevent harm and promote resiliency. While this function enhances adaptation and survival, deficiencies in safety learning in humans have been associated with profound alterations in behavior in stress-related disorders (Jovanovic et al., 2012; Laing and Harrison, 2021; Friedman et al., 2011). However, the mechanisms by which stress affects safety learning still remain elusive and highly neglected, while more attention has been given to how stress affects other related phenomena, such as facilitation of threat learning, impairment of cue and context discrimination, and failure in extinction learning (Conrad et al., 1999; Baratta et al., 2007; Holmes and Wellman, 2009; Dunsmoor and Paz, 2015; Maren and Holmes, 2016; Asok et al., 2018).

In a recent study, we implemented a novel approach to demonstrate that safety learning is highly susceptible to stress (Felix-Ortiz et al., 2024). Briefly, after undergoing social isolation stress for nearly two weeks, mice were exposed to a box arena containing multiple noxious cold quadrants (5°C, “threat zones”) and a more comfortable warm quadrant (30°C, “safety zone”). While visual cues differentiated these quadrants, ∼83% of the mice in the control group exhibited optimal memory for the thermal safety zone, whereas ∼67% of the mice in the stress group exhibited robust impairment of memory for the safety zone. Despite this striking finding, other important issues were not examined in our previous study. First, we did not sufficiently scrutinize the impacts of stress on stereotypical defensive behavior (Blanchard et al., 1986; LeDoux and Daw, 2018). Second, the previous study primarily considered male mice. Therefore, further investigation is needed to dissect potentially distinct impacts of stress in males and females on safety-seeking and defensive behavior (Luine et al., 2017; Merz and Wolf, 2017).

Emerging evidence suggests that male and female rodents could implement distinct behavioral strategies to deal with threat and adversity (Shansky, 2018, 2024; Foilb et al., 2021; Krueger and Sangha, 2021). Furthermore, female rodents often show more susceptibility to stress than males, especially during paradigms involving threat (Velasco et al., 2019; Day and Stevenson, 2020; Sangha et al., 2020; Furman et al., 2022). For instance, females exhibit stronger contextual threat conditioning and context generalization than males (Keiser et al., 2017). Females also exhibit more cue generalization than males during threat conditioning tasks involving CS+/CS- discrimination (Day et al., 2016). These observations are consistent with the fact that human female subjects exhibit greater risk for developing stress and trauma-related disorders (Kessler et al., 2012; Bangasser and Valentino, 2014; Shansky, 2015).

The present study implemented a comparative design with balanced samples for male and female mice to examine stress-related alterations in safety-seeking and stereotyped defensive behavior. While mice were subjected to our spatial-based thermal task after social isolation, three major categories were considered for defensive behavior. The first category included active defensive mechanisms such as rearing, darting, and jumping, which represent escape-related behaviors (Bolles, 1969; Gruene et al., 2015). The second category included passive defensive mechanisms such as freezing, crouching, and stretched posture, which represent protection, coping, and ambivalence, respectively (Van der Poel, 1967; Blanchard and Blanchard, 1969). The third category included other behaviors of relevance, such as grooming, general locomotion, and risk-taking (Spruijt et al., 1992; Ferreira et al., 2022). In summary, while stress altered safety memory and the relationships of safety-seeking behavior with other defensive mechanisms, there were no major sex differences in these effects.

## Materials and methods

### Subjects

A total of 64 C57BL6/J young adult mice (10-weeks-old, 32 males and 32 females) were obtained from a commercial source (The Jackson Laboratory). Upon arrival to our local vivarium, mice were housed in groups of four with food and water *ad libitum*, in a room with regulated temperature, pressure, and a regular 12-h light-dark cycle. After a week of acclimation, mice were brought into the laboratory space to undergo ear punching for identification and multiple handling sessions to become accustomed to human interactions. All animal procedures were conducted after approval by the Institutional Animal Care and Use Committee, in accordance with the Guide for the Care and Use of Laboratory Animals and the US Public Health Service’s Policy on Humane Care and Use of Laboratory Animals.

### Social isolation stress

As in our previous study (Felix-Ortiz et al., 2024), a social isolation stress procedure was implemented to evaluate the impacts of stress in safety learning and the structure of defensive behavior in male and female mice. Social isolation represents a major non-invasive form of stress that is capable of producing profound impacts on mood, affect, cognition, and behavior (Ieraci et al., 2016; Mumtaz et al., 2018; Lee et al., 2021). For this, individual cages containing four mice each (all males or all females) were randomly assigned to the control or stress groups. The no-stress controls (“CTL”) were always kept in group-housing conditions, whereas the social isolation stress groups (“SIS”) underwent single-housing conditions for a period of 14 days, after which they were regrouped with their original cagemates. Bodyweights were recorded every other day to evaluate the effectiveness of social isolation as a stressor that impairs weight gain (Arias et al., 2010; Mumtaz et al., 2018). Further validation of the social isolation stress procedure was achieved through assessment in the elevated-plus maze and open-field assays, which often reveal stress-induced anxiety-like behaviors (Calhoon and Tye, 2015). The plus-maze and open-field assays were conducted one day before and one day after the social isolation procedure. Seven days after the end of social isolation, mice underwent training and testing in the thermal safety task.

### Elevated-plus maze

The elevated-plus maze test was used to evaluate the effectiveness of the social isolation stress treatment. The test was conducted in a plus-shaped apparatus that consisted of two open arms (L:30 cm × W:5 cm) and two enclosed arms (L:30 cm × W:5 cm × H:15 cm) that intersected at a small central zone (L:5 cm × W:5 cm). The apparatus was significantly elevated from the benchtop (H:40 cm) and constructed from a beige-colored ABS plastic material (San Diego Instruments). The test was performed twice (10 min each time), one day before and one day after the social isolation treatment (i.e., pre-stress vs. post-stress testing). Since this strategy could lead to reductions in general exploration due to decreased contextual novelty during the second test, the pre-stress and post-stress tests were conducted in distinct procedure rooms with distinct overall contextual settings (e.g., different odors, different light patterns, different lab coats worn by the experimenters), in a counterbalanced fashion across mice to minimize such reductions. The time that mice spent in the open arms of the plus-maze apparatus was evaluated as a proxy of anxiety-like behavior (Walf and Frye, 2007). Significant reductions in open-arm exploration often emerge after stress exposure (Campos et al., 2013).

### Open-field test

The open-field test was performed to evaluate potential alterations in general locomotion by the social isolation stress treatment. The open-field assay was conducted in square-shaped transparent acrylic boxes (L:40 cm × W:40 cm × H:40 cm; Marketing Holders). Similar to the plus-maze assay, the open-field test was performed twice (10 min each time), one day prior to the beginning of social isolation and once again one day after the end of the social isolation treatment (i.e., pre-stress vs. post-stress testing). To minimize reductions in exploratory behavior due to decreased contextual novelty, the pre-stress and post-stress tests were conducted in distinct procedure rooms with distinct odors and illumination patterns in a counterbalanced fashion across mice. The average speed of locomotion was quantified using software as mice navigated the open-field arena. Significant reductions in locomotor activity during the open-field test could represent alterations in exploratory behavior or depressed states, which are common after stress (Yan et al., 2010; Seibenhener and Wooten, 2015).

### Thermal safety task

The impacts of social isolation stress on safety learning and the structure of defensive behavior were evaluated using a spatial-based thermal threat task that we recently developed (Felix-Ortiz et al., 2024). The task consisted of exposing mice to a square-shaped arena in which three quadrants had a significantly noxious cold temperature (∼5°C, “threat zones”), whereas the remaining quadrant had a more comfortable warm temperature (∼30°C, “safety zone”). These temperatures are consistent with previous reports of adverse and preferred temperatures, respectively, in mice (Gordon et al., 1998; Bautista et al., 2007). The cold and warm temperatures were achieved by placing ice or handwarmers underneath the floor. The stability of the temperatures was monitored using a digital thermometer gun (LaserPro LP300; Kizen) and a thermal imaging camera (C5; FLIR). Readjustments to the temperatures were made in between animals as needed. The task was conducted in transparent acrylic boxes (L:30 cm × W:30 cm × H:30 cm; Marketing Holders). These boxes were placed inside sound-attenuating cabinets (L:70 cm × W:86 cm × H:56 cm; Med Associates), which were equipped with fans that provided constant airflow and background noise (65 dB) to reduce distractions from external noise. The cabinets also reduced the role of distal visual cues within the procedure room to modulate spatial learning, an effect shown in our previous study (Felix-Ortiz et al., 2024). The cabinets were also equipped with regular white lights, near-infrared lamps, and overhead infrared cameras for the recording of videos. Proximal visual cues were added to the walls of the acrylic boxes to facilitate spatial learning. For example, plus symbols were paired with the warm zone, whereas vertical bars were paired with the cold zones. These visuospatial cues were counterbalanced across mice. The task consisted of two sessions. The training session lasted 10 min and included the warm and cold zones. In contrast, the recall test (conducted 24 h after training) lasted 5 min and only included a uniform cold temperature throughout the floor. The rationale for this was that if mice truly learned the precise location of the warm zone during training, the next day, they should still perform greater safety-seeking behavior within the correct zone even in the absence of the warm reinforcer, as validated in our previous study (Felix-Ortiz et al., 2024).

### Data analysis

All behavioral sessions were recorded at 15 fps. Mouse behavior was later quantified using two methods. The first method consisted of automated tracking of mice using software (ANYmaze; Stoelting). This method was implemented to measure various behaviors during the thermal task, including (a) Safety-seeking behavior, which was defined as the total time that mice spent within the spatial zone that predicted the warm temperature; (b) Freezing behavior, defined as periods of minimal mobility for at least half a second; (c) Risk-taking behavior, defined as periods in which mice protruded the head into the threat zones while keeping the rest of the body within the safety zone; and (d) General locomotion, assessed as the average speed of motion exhibited during a given behavioral session. Software-based quantifications were also implemented during the elevated-plus maze to measure the amount of time that mice spent in the open arms of the apparatus and during open-field testing to measure locomotion.

The second method used for behavioral quantifications consisted of software-assisted continuous hand-scored sampling of behavioral events. This method is still regarded as a highly validated strategy to quantify animal behavior (Altmann, 1974; Oldfield, 2001; Hämäläinen et al., 2016; Brereton et al., 2022), and was implemented by experimenters who were blind to mouse sex and treatment, similar to previous studies (Lehner, 1979; Felix-Ortiz et al., 2016). The following behaviors during the thermal task were quantified using this method: (a) Rearing behavior, defined as periods in which mice adopted an upright standing posture with their forelimbs touching the sidewalls of the box; (b) Darting behavior, defined as episodes in which mice suddenly made flight-like running moves; (c) Jumping behavior, which consisted of sudden hopping or leaping moves; (d) Crouching behavior, defined as periods in which mice adopted a hunched posture with the head and forelimbs rolled inward while supporting the weight of the body on the hindlimbs; (e) Grooming behavior, defined as sequences of self-cleaning behavior; and (f) Body stretching, defined as periods of low mobility while mice exhibited an elongated body posture.

An important correction was made between two of the measurements. While crouching behavior was hand-scored, freezing behavior was scored by the software. These behaviors are somewhat related to each other and most likely were quantified together by the software (Blanchard and Blanchard, 1969; Anagnostaras et al., 2010). To distinguish these two measurements, the total number of crouching episodes was subtracted from the total number of freezing episodes to correct the latter quantification.

Raw data was extracted from the software (ANYmaze) and tabulated using spreadsheets (MS Excel). After data analysis, graphs were generated, and the results were further evaluated using statistical software (GraphPad Prism). Each behavior was compared across the male, female, no-stress, and stress groups, as well as across behavioral sessions. Due to differences in the length of the training and recall sessions (10 min for training and 5 min for recall), all behaviors were analyzed in normalized forms (e.g., percent time in the safety zone, event rates per minute, or average speed of motion). The normality of the data was verified using the Kolmogorov-Smirnov test. Consistently, all results were plotted as mean ± standard error of the mean, with values for individual mice illustrated as scattered plots over the bar graphs. Significant differences across groups and sessions were evaluated using two-way repeated measures analysis of variance (ANOVA), with Holm-Šídák *post-hoc* tests for more statistical power (Salkind, 2007). Multiplicity-adjusted *P*-values were always considered to account for multiple comparisons. Chi-square tests were used to compare categorical proportions of mice that exhibited optimal memory versus poor memory, or stress-induced susceptibility versus stress resiliency (McHugh, 2013). Linear regression analysis was implemented to evaluate relationships among the distinct behaviors (Sheskin, 2003; Kleinhappel et al., 2019). For statistical significance, various two-tailed alpha levels were considered: *P < 0.05, **P < 0.01, ***P < 0.001, or ****P < 0.0001.

### Availability of data and materials

All the materials used in this study were obtained from commercial sources, as indicated throughout the methods. Software code and data will be shared for scientific use upon reasonable request.

## Results

### Overall experimental design to evaluate the effects of stress in safety learning

**FIGURE 1:**
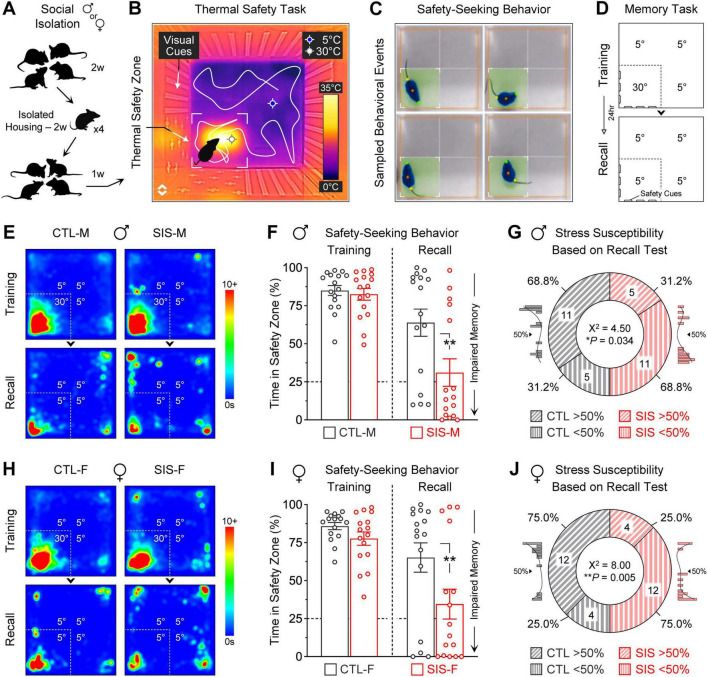
Social isolation stress led to robust impairment in safety memory, independent of mouse sex. **(A)** Depiction of a social isolation stress procedure. While controls remained in group-housing conditions, the experimental groups underwent single-housing for 14 days. After the isolation period, mice were returned to group-housing conditions with their former cagemates for a week, and then underwent training and testing in a safety learning task that involved a thermal threat. **(B)** Infrared image of the arena used for the thermal task, in which most quadrants had a noxious cold temperature (∼5°C, “threat zones”), while one quadrant had a pleasant warm temperature (∼30°C, “safety zone”). Visuospatial cues on the walls helped mice to differentiate the safety zone from the threat zones (e.g., plus symbols vs. vertical bars, counterbalanced across mice). **(C)** Examples of safety-seeking behavior. Software was used to track animals and quantify the time spent in the safety zone. **(D)** Experimental design to test for learning and memory. The training session lasted 10 min and included a warm safety zone, while the subsequent recall test lasted 5 min and no longer included the thermal reinforcer for the safety zone. **(E)** Average heatmaps for the male groups during the thermal task. **(F)** Quantifications of safety-seeking behavior for the male groups. Despite robust initial learning, many of the males that received the social isolation stress treatment exhibited significant memory impairment for the safety zone (**P = 0.0025). **(G)** Proportion of males that exhibited stress susceptibility or resiliency, based on memory recall. Consistent with the male distributions shown in the histogram insets, the cutoff was set to 50%. **(H)** Average heatmaps for the female groups during the thermal task. **(I)** Quantifications of safety-seeking behavior for the female groups. Similar to males, many of the females that received the social isolation stress treatment exhibited significant memory impairment for the safety zone (**P = 0.0086). **(J)** Proportion of females that exhibited stress susceptibility or resiliency, based on memory recall. Consistent with the female distributions shown in the histogram insets, the cutoff was set to 50% (*N* = 16 per group; CTL, no-stress control; SIS, social isolation stress; M, males; F, females).

We recently showed that a two-week social isolation stress period was highly detrimental for the formation of lasting representations of safety when exposed to a box containing thermal threat and safety zones (Felix-Ortiz et al., 2024). Since our previous study primarily considered male mice, the first goal of the present study was to determine whether the effects of this stressor are sex-specific or sex-independent. We implemented a comparative design that included balanced samples of male and female mice (*N* = 32 for each sex). Initially, these mice were separated by sex and were housed in groups of four for approximately two weeks. Then, the cages were randomly assigned to either the control or experimental groups. While the control groups were kept in group-housing conditions (i.e., no-stress, CTL-M or CTL-F; *N* = 16 per sex), mice in the experimental groups underwent a two-week isolated-housing period (i.e., social isolation stress, SIS-M or SIS-F; *N* = 16 per sex). After the two-week isolation period, the stressed mice were regrouped with their former cagemates (Figure 1A). One week later, all mice underwent training in the thermal task in which the behavioral arena had three noxious cold zones and a comfortable warm zone (Figure 1B). The warm zone was differentiated from the cold zones using discrete visuospatial cues on the wall of the apparatus. As in our previous study, the most prominent behavior exhibited by mice during training was safety-seeking, which was evaluated as total time spent within the warm zone (Figure 1C). The next day, mice underwent a test session in the absence of the warm temperature to evaluate long-term recall of the safety memory (Figure 1D). During the recall test, based on the position of the visuospatial cues on the walls, biased seeking behavior toward the zone that previously predicted thermal safety was indicative of optimal memory (Felix-Ortiz et al., 2024).

### Validation of the social isolation treatment as a potent stressor

The effectiveness of the two-week social isolation treatment as a stressor was evaluated in a subset of mice (*N* = 8 per sex per treatment, i.e., half of the controls and half of the stressed animals) (Supplementary Figure 1A). Three methods of evaluation were implemented: (a) Changes in bodyweight gain, (b) Changes in exploration patterns during elevated-plus maze testing, and (c) Changes in locomotor activity during open-field testing. Bodyweights were assessed every other day during the social isolation period. The plus-maze and open-field assays were conducted twice, one day before and one day after the social isolation period. To minimize reductions in exploratory behavior due to decreases in contextual novelty, these assays were performed in counterbalanced manners in distinct procedure rooms.

The social isolation treatment significantly delayed the progression of bodyweight gain in both sexes. While the male groups exhibited a significant group × time interaction [Supplementary Figure 1B, Top Panel; *F*_(7_, _210)_ = 3.25, *P* = 0.0027], the female groups also exhibited a significant group × time interaction [Supplementary Figure 1B, Bottom Panel; *F*_(7_, _210)_ = 2.50, *P* = 0.018]. In addition, the social isolation treatment produced significant reductions in open-arm exploration during plus-maze testing. While both male groups exhibited reductions when comparing the first and second plus-maze tests, only the reduction in the stress-male group was significant [Supplementary Figure 1C, Top Panel; Time Factor, *F*_(1_, _14)_ = 17.5, *P* = 0.0009; *post-hoc* test for CTL-M, *P* = 0.21; *post-hoc* test for SIS-M, *P* = 0.0054]. Similarly, while both female groups exhibited reductions when comparing the first and second plus-maze tests, only the reduction in the stress-female group was significant [Supplementary Figure 1C, Bottom Panel; Time Factor, *F*_(1_, _14)_ = 15.2, *P* = 0.0016; *post-hoc* test for CTL-F, *P* = 0.23; *post-hoc* test for SIS-F, *P* = 0.011]. Importantly, no significant alterations were observed in general locomotion during open-field testing for the male groups [Supplementary Figure 1D, Top Panel; Interaction Factor, *F*_(1_, _14)_ = 0.44, *P* = 0.52] or the female groups [Supplementary Figure 1D, Bottom Panel; Interaction Factor, *F*_(1_, _14)_ = 3.34, *P* = 0.89]. Collectively, these results are consistent with well-established effects of stress, considering social isolation and other types of stressors, thereby validating once again the social isolation paradigm as a significant stressor that alters behaviors associated with internal states, such as anxiety (Walf and Frye, 2007; Mumtaz et al., 2018).

### The stress treatment was highly detrimental for safety memory in both sexes

Males exhibited robust stress-induced impairments in safety memory during the thermal task. During the training session, similar to the control-male group, the stress-male group showed high levels of safety-seeking behavior (Figures 1E, F; Training Session, CTL-M vs. SIS-M, 85 vs. 83% time spent in the safety zone; *P* = 0.80). However, during the subsequent recall test, while the control-male group showed relatively high levels of safety-seeking behavior, the stress-male group showed significantly lower levels of safety-seeking behavior (Figures 1E, F; Recall Session, CTL-M vs. SIS-M, 64 vs. 31% time spent in the safety zone; *P* = 0.0025). Additional analysis of the individual data revealed that while many control-males showed good safety recall (11/16, 69%), some control-males showed poor safety recall (5/16, 31%). In contrast, while many stress-males showed poor safety recall (11/16, 69%), some stress-males showed good safety recall (5/16, 31%). A categorical chi-square test comparing these ratios revealed a significant difference between the control-male and stress-male groups (Figure 1G; *X*^2^ = 4.50, *P* = 0.034). These findings are consistent with our previous study.

Females also exhibited robust impairments in safety memory during the thermal task. As in the male groups, the control-female and stress-female groups showed high levels of safety-seeking behavior during the training session (Figures 1H, I; Training Session, CTL-F vs. SIS-F, 86 vs. 78% time spent in the safety zone; *P* = 0.43). Despite this, during the subsequent recall test, while the control-female group showed relatively high levels of safety-seeking behavior, the stress-female group showed significantly lower levels of safety-seeking behavior (Figures 1H, I; Recall Session, CTL-F vs. SIS-F, 65 vs. 35% time spent in the safety zone; *P* = 0.0086). Furthermore, the individual data revealed subsets of control-females showing good safety recall (12/16, 75%), subsets of control-females showing poor safety recall (4/16, 25%), subsets of stress-females showing poor safety recall (12/16, 75%), and subsets of stress-females showing good safety recall (4/16, 25%). A categorical chi-square test comparing these ratios revealed a significant difference between the control-female and stress-female groups (Figure 1J; *X*^2^ = 8.00, *P* = 0.005). Thus, the social isolation treatment produced similar susceptibility in males and females for the formation of lasting representations of safety.

### Active defensive mechanisms related to escape were unaffected by the stress treatment

Our next goal was to determine whether the stress treatment affected other behaviors that could potentially explain the impairments observed during the task. After inspecting a few representative videos, we realized that in addition to safety-seeking behavior, mice often exhibited multiple behaviors related to escape, including rearing, darting, and jumping. These behaviors represent active defensive mechanisms that help animals avoid harm as they experience threat (Bolles, 1969; Claudi et al., 2021). Therefore, a scoring method was implemented to quantify these behaviors for all animals and sessions and to perform comparisons across the control, stress, male, and female groups.

**FIGURE 2:**
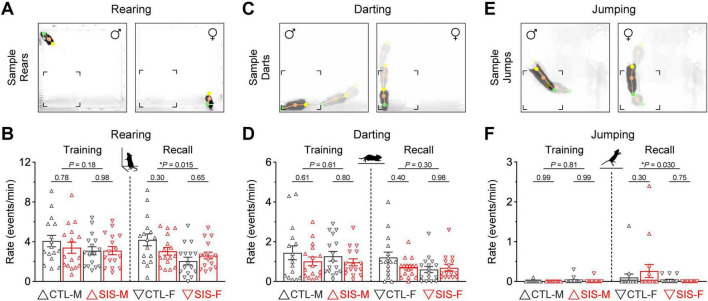
Active defensive mechanisms related to escape were strongly exhibited during the thermal safety task. **(A)** Rearing behavior, quantified when mice adopted an upright standing posture with forelimbs touching the sidewalls of the box. **(B)** Rearing behavior did not differ between the control and stress groups. However, males exhibited significantly more rearing than females during the recall test (*P < 0.05). **(C)** Darting behavior, defined as events in which mice made sudden flight-like running movements in any direction. Representative events are shown as overlaid video frames. **(D)** The rate of darting behavior did not differ between the control and stress groups, or the male and female cohorts. **(E)** Jumping behavior, defined as events in which mice exhibited sudden hopping or leaping movements. Representative events are shown as overlaid video frames. **(F)** While only a handful of mice exhibited jumping behavior, no significant differences were detected when comparing the control and stress groups. However, males tended to do more jumping than females, particularly during the recall test (*P < 0.05) (*N* = 16 per group; CTL, no-stress control; SIS, social isolation stress; M, males; F, females).

Rearing behavior was defined as periods in which mice adopted an upright standing posture, supporting their body on the hindlimbs while touching the sidewalls of the box with the forelimbs (Figure 2A). Rearing was the most frequent escape-related behavior during both phases of the thermal task (Figure 2B). While the rate of rearing behavior did not differ between the control and stress groups (stats in graph), males exhibited significantly more rearing behavior than females, but only during the recall test (MvsF; Training, *P* = 0.18; Recall, *P* = 0.015).

Darting behavior was defined as mice making sudden flight-like running moves in any direction from anywhere in the box (Figure 2C). While not as frequent as rearing, most animals exhibited some degree of darting, regardless of sex or treatment during both phases of the experiment (Figure 2D). The rate of darting behavior did not differ between the control and stress groups (stats in graph). Darting also did not differ between the male and female cohorts (MvsF; Training, *P* = 0.61; Recall, *P* = 0.30).

Jumping behavior was defined as mice performing sudden hopping or leaping moves anywhere in the box (Figure 2E). This was the least frequent escape-related behavior and was primarily exhibited by only a few animals (Figure 2F). While jumping was unaffected by stress (stats in graph), males tended to display more jumping behavior than females during the recall test (MvsF; Training, *P* = 0.81; Recall, *P* = 0.03). Thus, these active defensive mechanisms related to escape seemed to be unaffected by the stress treatment, despite some minor (yet statistically significant) sex differences.

### Passive defensive mechanisms were also unaffected by the stress treatment

Next, we evaluated possible stress-induced alterations in passive defensive mechanisms. This included behaviors such as freezing, crouching, and stretched postures. These behaviors have been associated with various functions, including protection, coping, and ambivalence during threat and adversity (Blanchard and Blanchard, 1969; Blanchard et al., 1986; Kaesermann, 1986).

**FIGURE 3:**
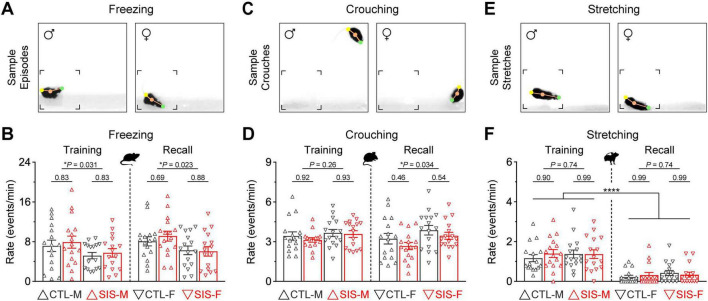
Passive defensive mechanisms related to protection, coping, and ambivalence were also prominent during the thermal safety task. **(A)** Freezing behavior, defined as periods of minimal mobility. This behavior is often implemented as a protection mechanism against threat, pain, or punishment. **(B)** The rate of freezing behavior did not differ between the control and stress groups. However, males exhibited significantly more freezing than females during both sessions (*P < 0.05). **(C)** Crouching behavior, defined as periods in which mice adopted a hunched posture with the head and forelimbs rolled inward while supporting most of the body on the hindlimbs. This behavior is often implemented as a coping mechanism against cold temperatures. **(D)** The rate of crouching behavior did not differ between the control and stress groups. However, females exhibited significantly more crouching than males during the recall test (*P < 0.05). **(E)** Body stretching, defined as periods in which mice exhibited an elongated body posture. Stretched postures in rodents represent periods of ambivalence during threat, conflict, or uncertainty. **(F)** Body stretching was exhibited more prominently during the training session in all groups (****P < 0.0001). Yet, this behavior did not differ between the control and stress groups, or the male and female cohorts, during either training or recall (*N* = 16 per group; CTL, no-stress control; SIS, social isolation stress; M, males; F, females).

Freezing behavior in mammals offers protection against threat in the environment (LeDoux and Daw, 2018). In this study, freezing behavior was defined as periods of minimal mobility for at least half a second (Figure 3A), which is consistent with other studies implementing quantifications for this behavior (Anagnostaras et al., 2010; Burgos-Robles et al., 2017). This behavior was the most frequent passive mechanism exhibited by animals during the thermal task (Figure 3B). While the rate of freezing episodes did not differ between the control and stress groups (stats in graph), males tended to exhibit more freezing behavior than females during both phases of the experiment (MvsF; Training, *P* = 0.031; Recall, *P* = 0.023). Sex differences in freezing behavior are consistent with previous observations during other behavioral tasks (e.g., Gruene et al., 2015; but see Borkar et al., 2020).

Crouching behavior was defined as periods in which mice adopted a hunched posture with the head and forelimbs rolled inward while supporting most of the body on the hindlimbs (Figure 3C). Warm-blooded animals often implement this behavior as a coping mechanism to regulate body temperature during exposure to cold (Chappell and Holsclaw, 1984). Mice of both sexes exhibited crouching behavior quite frequently during the thermal task (Figure 3D). While the rate of crouching did not differ between the control and stress groups (stats in graph), females tended to exhibit more crouching than males during the recall test (MvsF; Training, *P* = 0.26; Recall, *P* = 0.034).

Stretched body postures were also exhibited during the thermal task (Figure 3E). This passive defensive mechanism was not as frequent as freezing or crouching but was more prominent during the training session (Figure 3F; Training vs. Recall, *P* < 0.0001 for all groups). Yet, the rate of stretched postures was unaffected by stress (stats in graph), and no significant differences were observed between males and females (MvsF; Training, *P* = 0.74; Recall, *P* = 0.74). Thus, similar to the active mechanisms examined above, these passive defensive mechanisms seemed unaltered by the stress treatment despite minor (yet statistically significant) differences between males and females.

### Other behaviors of interest were also insensitive to the stress treatment

Additional behaviors of interest during the thermal task were also evaluated for potential impact by stress. These included risk-taking, self-grooming, and general locomotion. Changes in risk-taking could represent alterations in decision-making processes (Laviola et al., 2003; Dent et al., 2014; Friedman et al., 2017). Self-grooming in rodents has been associated with many biological functions beyond cleanliness, including pain relief (Vos et al., 1998) and thermoregulation (Thiessen, 1988). Furthermore, alterations in self-grooming have been considered as behavioral markers of emotional distress, especially during situations involving conflict (Tinbergen, 1952; Rojas-Carvajal and Brenes, 2020). Changes in locomotion could represent alterations in mood, affect, or exploratory behavior (Yan et al., 2010; Seibenhener and Wooten, 2015).

**FIGURE 4:**
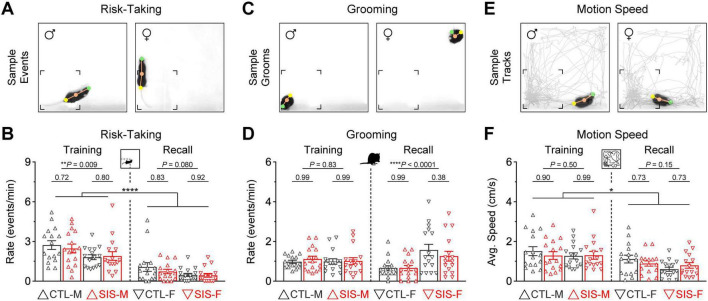
Other behaviors of interest during the thermal task. **(A)** Risk-taking behavior, defined as periods in which mice protruded their head into the cold zones while keeping the rest of the body inside the warm zone. **(B)** Risk-taking was exhibited more prominently during the training session in all groups (****P < 0.0001). While risk-taking did not differ between the control and stress groups, males exhibited significantly more risk-taking than females during the training session. **(C)** Grooming behavior as an index of stress during exposure to the cold. **(D)** Grooming rates did not differ between the control and stress groups. However, females exhibited a lot more grooming than males during the recall test (****P < 0.0001). **(E)** Motion speed as an index of general locomotion. **(F)** Although all the groups exhibited slightly faster average speeds during the training session than the recall test (*P < 0.05), the motion speeds did not differ between the control and stress groups, or the male and female cohorts (*N* = 16 per group; CTL, no-stress control; SIS, social isolation stress; M, males; F, females).

Risk-taking during the thermal task was defined as periods in which mice protruded their head into the cold zones while keeping the rest of the body inside the warm zone (Figure 4A). This behavior was notably more prominent during the training session (Figure 4B; Training vs. Recall, *P* < 0.0001 for all groups). While this behavior did not differ between control and stress (stats in graph), male mice exhibited significantly more risk-taking than the female mice (MvsF; Training, *P* = 0.009; Recall, *P* = 0.08). This is consistent with previous observations in which male rodents are more willing to perform behaviors that involve higher risk, whereas female rodents are more risk-averse and prefer to behave in a manner that involve lower risk (Orsini et al., 2016; Orsini and Setlow, 2017; Gomes et al., 2022; Shi et al., 2023).

Grooming was quantified when mice performed stereotyped behavior related to self-cleaning, including licking of the forelimbs, nose, face, head, or other parts of the body (Figure 4C). Grooming sequences were exhibited in similar amounts during both phases of the task (Figure 4D). While the rate of grooming events did not differ between the control and stress groups (stats in graph), females exhibited significantly more grooming behavior than males during the recall test (MvsF; Training, *P* = 0.83; Recall, *P* < 0.0001). This is inconsistent with prior studies showing that male rodents exhibit more self-grooming behavior than females during novelty or threat tasks (Thor et al., 1988; Borkar et al., 2020).

Finally, general locomotion was defined as the average speed of motion during each session (Figure 4E). All groups showed slightly higher motion speeds during the training session (Figure 4F; Training vs. Recall, *P* < 0.05 in all groups). However, motion speed did not differ between the control and stress groups (stats in graph), or between the male and female groups (MvsF; Training, *P* = 0.50; Recall, *P* = 0.15). Therefore, similar to the other defensive mechanisms examined above, these additional behaviors seemed unaltered by the stress treatment and could not explain the profound alterations observed in the formation of lasting representations for the safety zone.

### Further analysis revealed that the stress treatment disorganized defensive behavior

**FIGURE 5:**
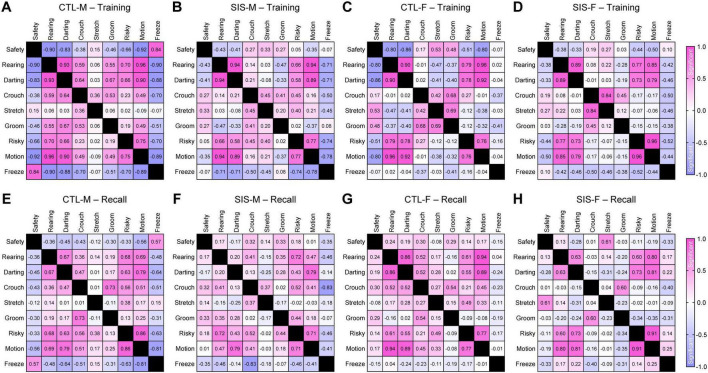
Linear regressions amongst the behaviors. **(A–D)** Regressions during the training session. **(E–H)** Regressions during the recall test. Correlations were computed for all the behavioral pairs, except for jumping behavior due to undersampling. These analyses considered Gaussian distributions, sixteen sample per correlation, and two-tailed *P*-values. Correlation coefficients greater than ±0.50, ±0.62, ±0.75, or ±0.82 corresponded to statistical significance levels of *P* < 0.05, *P* < 0.01, *P* < 0.001, or *P* < 0.0001, respectively. Graphical representations for all the linear regressions are shown in Supplementary Figures 2–4 (CTL, no-stress control; SIS, social isolation stress; M, males; F, females).

Despite the stress treatment not impacting the overall quantity of defensive behavioral episodes, the final objective of this study was to investigate whether stress altered potential interactions and relationships among the behaviors. To achieve this, we implemented pairwise linear regression analysis considering all of the recorded behaviors, except for jumping which showed very low frequencies and would have skewed the analysis due to undersampling. Then, the correlation coefficients were plotted into correlation matrix as heatmaps, using red-shifted colors for positive correlations and blue-shifted colors for negative correlations (Figure 5). Based on sixteen pair-wise samples, correlation coefficients greater than ±0.50, ±0.62, or ±0.75 corresponded to statistical significance levels of *P* < 0.05, *P* < 0.01, or *P* < 0.001, respectively.

In males, various significant relationships were detected between safety-seeking and other defensive mechanisms, especially during the training session in the control group but not in the stress group. For instance, safety-seeking was negatively correlated with rearing, darting, and risk-taking in the control-male group (Figure 5A; Safety vs. Rearing, *R* = −0.90, *P* < 0.0001; Safety vs. Darting, *R* = −0.83, *P* < 0.0001; Safety vs. Risk-Taking, *R* = −0.66, *P* = 0.006). In contrast, these behaviors were uncorrelated in the stress-male group (Figure 5B; Safety vs. Rearing, *R* = −0.43, *P* = 0.095; Safety vs. Darting, *R* = −0.41, *P* = 0.12; Safety vs. Risk-Taking, *R* = 0.05, *P* = 0.86). In addition, safety-seeking was positively correlated with freezing behavior in the control-male group (Figure 5A; Safety vs. Freezing, *R* = 0.84, *P* < 0.0001), whereas these behaviors were uncorrelated in the stress-male group (Figure 5B; Safety vs. Freezing, *R* = −0.07, *P* = 0.81). Thus, the stress treatment seemed to affect the organization of these behaviors.

Similar to males, the female groups exhibited significant correlations of safety-seeking, rearing, darting, and risk-taking during training in the control group but not in the stress group. That is, safety-seeking was negatively correlated with rearing, darting, and risk-taking in the control-female group (Figure 5C; Safety vs. Rearing, *R* = −0.80, *P* = 0.0002; Safety vs. Darting, *R* = −0.86, *P* < 0.0001; Safety vs. Risk-Taking, *R* = −0.51, *P* = 0.044). In contrast, these behaviors were uncorrelated in the stress-female group (Figure 5D; Safety vs. Rearing, *R* = −0.38, *P* = 0.15; Safety vs. Darting, *R* = −0.33, *P* = 0.21; Safety vs. Risk-Taking, *R* = −0.44, *P* = 0.09). In contrast to males, safety-seeking and freezing behavior were not correlated in either the control-female group (Figure 5C; Safety vs. Freezing, *R* = −0.07, *P* = 0.79) or the stress-female group (Figure 5D; Safety vs. Freezing, *R* = 0.10, *P* = 0.73). Such sex difference could be attributed to the observation of significantly lower freezing rates in females than males.

Unlike the training session, during the memory recall test, safety-seeking showed limited correlations with the other defensive behaviors in all groups (Figures 5E–H). Moreover, some behaviors exhibited very stable relationships across all groups during both sessions (e.g., rearing and darting). Finally, crouching and stretched postures showed very limited correlations or did not exhibit clear patterns of effects by stress during either of the sessions. Collectively, these findings suggest that stress may have reorganized specific defensive behaviors, such as rearing, darting, and risk-taking, in a manner that mice were still capable of exhibiting these behaviors, but they were exhibited in chaotic manners in relationship to safety-seeking. Disorganized defensive behavior during training could represent a significant mechanism by which stress led to alterations in the formation of safety memory.

## Discussion

This study evaluated sex differences and the negative impacts of social isolation stress for memory formation during a behavioral task that involved safety-seeking and avoidance-related behaviors while mice were getting exposed to an environment containing thermal threat. Major focus was given to stress-induced alterations in active defensive mechanisms (e.g., darting, rearing, and jumping) and passive defensive mechanisms (e.g., freezing, crouching, and ambivalent posture), as well as in other behaviors relevant to the task (e.g., risk-taking, grooming, and locomotion). This represents a broad approach from which the results provided new insights into the behavioral mechanisms that potentially contributed to the stress-induced impairments that were observed in safety memory. In summary, the results showed that lasting representations for the safety quadrant were highly affected in male and female mice that underwent the social isolation stress procedure, but not in mice that always remained in group-housing conditions. Furthermore, while the stress treatment did not affect the average frequency of the defensive mechanisms that are listed above, the stress treatment seemed to reorganized the structure of several of the defensive mechanisms in such a way that they became uncorrelated with safety-seeking behavior. Overall, these findings highlight the severity of social isolation stress as a critical factor that contributes to significant alterations in behavioral mechanisms that facilitate resiliency, adaptability, and memory formation in the face of threat and adversity.

### Advantages for evaluating safety learning with a spatial-based task

Safety learning has been traditionally evaluated using “conditioned inhibition paradigms” in which a particular cue (e.g., a tone or light) is capable of diminishing fear-related responses to another cue that explicitly predicts a noxious stimulus (Kendrick, 1958; Odriozola and Gee, 2021). While improvements have been made over the years, some issues still remain unresolved for conditioned inhibition paradigms. For instance, while threat learning and memory are clearly represented by defensive behavioral responses such as freezing, safety learning and memory are often not represented by any particular behavior during conditioned inhibition paradigms, but instead safety is inferred from the reductions observed in freezing responses when the threat and safety cues are presented simultaneously (Donahoe and Palmer, 1988; Sosa and Ramírez, 2019). However, this is not the case for the spatial-based thermal safety task implemented in the present study, which we developed and thoroughly validated in a recent publication (Felix-Ortiz et al., 2024). In this task, mice get to develop actual safety-seeking behavior as they freely explore the open-field arena containing the distinct thermal zones that are paired with the noxious and pleasant temperatures (5 vs. 30°C, respectively). Importantly, these thermal zones are paired with visual cues on the walls of the apparatus that facilitate spatial orientation, navigation, and association with the particular temperatures. Recognition of these visual cues during the subsequent recall test can then guide distinctive behaviors associated to the threat and safety memories, such as avoidance and seeking, respectively. These clearly quantifiable behaviors render this spatial-based task particularly suitable for evaluating the mechanisms of safety learning and memory.

Threat avoidance and safety seeking are not the only quantifiable behaviors during the thermal safety task. The present study shows that a repertoire of defensive mechanisms are also exhibited by mice of both sexes during this task. Particularly during the training session, mice frequently exhibited behaviors related to escape (e.g., darting and rearing), coping (e.g., crouching and freezing), ambivalence (e.g., stretched posture), and risk-taking (e.g., head dipping). Although many of these behaviors were most likely related to innate or reflexive reactions to the noxious cold temperature, rather than learned behaviors (Blanchard et al., 1986; LeDoux and Daw, 2018), some of these behaviors were strongly correlated with safety-seeking behavior in both sexes. In addition, the social isolation stress treatment turned those correlations insignificant during the training session, despite good acquisition of safety-seeking behavior. Thus, this could represent a crucial mechanism by which the stress treatment rendered many of the male and female mice more susceptible to impairments in lasting representations and subsequent recall for the safety zone. Yet, further investigation is needed to determine how each defensive mechanism contributes to the formation of safety engrams. For instance, neural pathways that are critical for particular defensive behaviors could be individually targeted as mice undergo the thermal safety task to assess the functional role for individual behaviors. Following this logic, a neural pathway of interest could be the dorsal peduncular cortex to the central amygdala, which is necessary for flight-related escape responses during threat (Borkar et al., 2024).

### Comparisons of the thermal safety task with other relevant spatial-based paradigms

While this is not the first time that a research study has sought to evaluate behaviors associated with threat avoidance and safety seeking using a more ethologically-relevant spatial-based paradigm, the present task is distinctively characterized by the use of thermal reinforcers. Other tasks, for example, have implemented predator odors (e.g., cat saliva, bobcat urine, or fox trimethylthiazoline), which upon their detection, laboratory rodents immediately exhibit robust responses such as freezing and avoidance (Dielenberg and McGregor, 2001; Albrechet-Souza and Gilpin, 2019; Arakawa, 2019; Engelke et al., 2021). These behaviors are similar to the ones observed in the present task. However, tasks involving predator odor have not been traditionally used to evaluate safety learning. Another paradigm that is even more analogous to the present task is the traditional water maze. In the water maze task, rodents are required to swim until they find a submerged platform. As training progresses, rodents implement visuospatial learning strategies to navigate faster and more efficiently toward the location of the platform. Although the water maze has most often been regarded as a paradigm for studying spatial learning and memory (Morris, 1984; Brandeis et al., 1989; Othman et al., 2022), in principle the water maze is also a paradigm that evaluates safety learning and memory, similarly to the present safety task. While in the traditional water maze task rodents escape the zones with a deep-water level (i.e., danger) by seeking for the precise location that has been paired with a shallow-water level (i.e., safety), in the present thermal task rodents escape the zones with a noxious-cold temperature (i.e., danger) by seeking for the precise location that has been paired with a pleasant-warm temperature (i.e., safety). Notably, in both paradigms, safety-seeking behavior during memory tests is mainly driven by visuospatial cues. Due to these similarities, the present thermal safety task could also be regarded as a “dry version of the water maze.” Indeed, this idea has been proposed by previous studies that implemented similar thermal tasks to study place memory in crickets and drosophila flies (Wessnitzer et al., 2008; Ofstad et al., 2011). In any case, an advantage of the present thermal task is that it simply requires a relatively small open-field arena and some simple sources of cold and warm temperatures (e.g., ice and hand-warmers, respectively), as opposed to a much larger apparatus filled with water as in the traditional water maze.

### Sex differences in threat-related behavior and safety learning

During the thermal safety task, males exhibited more freezing behavior than females during both the training and recall sessions. Furthermore, males exhibited more rearing, jumping, and risk-taking behavior than females, whereas females exhibited more crouching and grooming than males during some of the sessions. These findings are consistent with growing evidence that male and female rodents implement distinct behavioral strategies when dealing with situations involving threat. For instance, during classical threat conditioning (i.e., cue predicts shock), while freezing behavior characterizes fear-related responses in males, females preferably exhibit darting behavior (Gruene et al., 2015; Clark et al., 2019; Mitchell et al., 2022). In contrast, newer versions of threat conditioning that allow transitions from freezing to darting have yielded observations that are somewhat contradictive, as males showed less freezing behavior than females (Borkar et al., 2020). Despite this, differences in the learning contingencies during distinct tasks could explain the discrepancies in behavioral sex differences. Nonetheless, future studies could implement approaches in which male and female animals undergo systematic testing in a variety of behavioral tasks to better understand sex-specific behavioral differences.

There is also some documented evidence for sex differences in safety learning, which has been mostly gathered using the cue discrimination and conditioned inhibition paradigms. In one study, female rats exhibited better discrimination of threat and safety cues than male rats. However, this sex difference did not result in better suppression of fear responses when the two cues were presented simultaneously (Foilb et al., 2018). In another study, while male rats exhibited substantial suppression of fear responses during simultaneous presentation of the threat and safety cues, female rats failed to exhibit significant reductions in fear responses (Krueger and Sangha, 2021). While somewhat contradictive, another study revealed that similar to male rats, females in metestrus/diestrus (i.e., low-estrogen phase) exhibited optimal discrimination of the threat and safety cues, whereas females in proestrus (i.e., high-estrogen phase) exhibited poor discrimination between these types of cues (Trask et al., 2020). Thus, it seems that sex differences during safety signaling could be related to variation in the estrous cycle (Peyrot et al., 2020; Shansky, 2024). While the present study did not consider the estrous cycle, future studies could consider careful evaluations of the estrous cycle and sex hormones for the thermal safety task.

### The influence of stress in threat-related behavior and safety learning

The influence of stress in safety learning remains largely underexplored compared to the impacts of stress in threat learning (Peyrot et al., 2020; Merz and Wolf, 2022). It is well established that after repeated restraint or immobilization stress, during traditional threat learning paradigms rodents exhibit significant enhancement in the acquisition of conditioned freezing responses and resistance to extinguish those responses (Conrad et al., 1999; Miracle et al., 2006; Wood et al., 2008; Hoffman et al., 2015; Maren and Holmes, 2016). Similar effects have been reported with other stressors, such as prolonged social isolation, repeated unsignaled footshocks, or chronic variable stress paradigms (Lukkes et al., 2009; Rau and Fanselow, 2009; Sanders et al., 2010; Hassien et al., 2020). In contrast to facilitation in threat learning, some significant impairments have been reported for safety learning after stress. For instance, forced swim and restraint stress have been shown to impair conditioned inhibition paradigms (Adkins et al., 2022; Wang et al., 2024). Furthermore, we recently showed that social isolation stress produces robust deficits in safety learning during thermal threat (Felix-Ortiz et al., 2024). The present study further expanded this finding by showing that male and female mice are equally vulnerable to the impacts of social isolation stress in safety learning. Furthermore, while the present study shows robust impairments in safety learning at seven days after ceasing social isolation, our previous study reported similarly robust impairments at fourteen days after ceasing social isolation (Felix-Ortiz et al., 2024). Collectively, these findings suggest that the neural substrates underlying safety learning are highly vulnerable to stress in general, and that stress-induced alterations in the neural substrates for safety learning persist for long periods. Ongoing studies by our group are further testing these ideas while considering multiple stressors.

### Sex differences in stress vulnerability during threat and safety learning

Growing evidence suggests that females are more susceptible to stress than males. For instance, female mice are more prone to bodyweight loss and the development of hyperactive, anxious-like behavior during chronic stress than males (Furman et al., 2022). These observations are consistent with the greater risk for stress and trauma-related disorders observed in human female subjects (Kessler et al., 2012; Gradus, 2017). In addition to the traditional view of stress-induced enhancement in threat learning, it seems that impairments in safety learning also contribute to greater risk for stress and trauma-related disorders, as observed in PTSD patients (Jovanovic et al., 2010, 2012). However, mixed results have been reported for females in preclinical studies implementing stress and conditioned inhibition paradigms (Day et al., 2016; Foilb et al., 2018; Clark et al., 2019; Krueger and Sangha, 2021; Adkins et al., 2022). In addition, the present study revealed that during the thermal safety task, female mice do not show greater susceptibility stress than males. Future studies could consider other factors, such as the estrous cycle as mentioned above, to further disentangle possible sex differences in the effects of stress during safety learning and memory (Trask et al., 2020).

### Implications of behavioral disorganization after stress

An intriguing finding in the present study is that while the stress treatment did not affect the frequency of threat-related defensive behavior, the stress treatment instead seemed to reorganize the structure of defensive behavior. This outcome was uncovered by the multi-regression analysis, which considered most of the behaviors assessed, and showed that while several behaviors such as rearing, darting, and risk-taking were highly correlated with safety seeking in the control groups, such correlations were not exhibited by the stress-exposed groups. It is important to highlight that these findings were particularly noted during the training session in which the control and stress groups did not differ in safety-seeking behavior. Yet, the stress groups subsequently exhibited robust impairments in safety seeking during the recall test. Collectively, these findings suggest that the stress treatment disorganized defensive behavior, which could be a critical mechanism by which the encoding of safety memory is altered after stress. This idea is consistent with observations in human patients that exhibit behavioral disorganization after profound stress and traumatic experiences. Indeed, disorganized behavior is part of the criteria for the diagnosis of post-traumatic stress disorder (PTSD), especially during early ages (Veenema and Schroeder-Bruce, 2002; American Psychiatric Association, 2013). In addition, PTSD is associated with memory loss and cognitive impairments during safety learning (Jovanovic et al., 2012; Straus et al., 2018; Keefe et al., 2022), extinction-based exposure therapy (Pitman et al., 2012; VanElzakker et al., 2014; Wicking et al., 2016), and when PTSD patients need to cope with new trauma (Morey et al., 2015; Maeng and Milad, 2017). In light of the present findings, perhaps these impairments in PTSD patients could be attributed to maladaptive coping strategies involving disorganized behavior.

## Concluding remarks

Despite the fact that stress produced similar effects in males and females, this study represents a significant step forward toward developing a better understanding for sex factors and the impacts of stress in safety learning. After considering at least ten behaviors during the safety task, our female subjects did not exhibit a greater impact of stress than the male subjects. Furthermore, additional measurements during the plus-maze and open-field tests also revealed no greater impacts of stress in the female subjects. Nonetheless, we only evaluated social isolation stress, and future studies could implement other types of stressors to either refute our results or confirm that female mice are indeed similarly vulnerable to stress than male mice with respect to safety learning.

## Data Availability

The original contributions presented in this study are included in this article/Supplementary material, further inquiries can be directed to the corresponding author.

## Supplementary material

Supplementary Figure 1 Validation of the social isolation procedure. **(A)** Depiction of timeline of the social isolation stress procedure. **(B)** Bodyweight measurements every other day during the social isolation procedure. Delays in bodyweight gain represent effectiveness of treatments as emotional stressors. **(C)** Behavioral testing in the elevated-plus maze (EPM) before and after the social isolation procedure. Significant decays in the time of open arm exploration represent effectiveness of treatments as emotional stressor. While the no-stress control groups showed no significant changes between the pre and post sessions (Males, *P* = 0.21; Females, *P* = 0.23), the stress groups exhibited significant decays in open arm exploration (Males, ***P* = 0.0054; Females, **P* = 0.011). **(D)** Traditional open-field test (OFT) before and after the social isolation procedure. No significant changes were detected for the average speed of motion in the open field (all *P*’s > 0.53) (*N* = 8 per group CTL, control; SIS, stress).

Supplementary Figure 2 Linear regressions comparing safety-seeking behavior and the active defensive mechanisms. **(A,B)** Relationships between rearing and safety-seeking behavior during the training and recall sessions. **(C,D)** Relationships between darting and safety-seeking behavior during the training and recall sessions. **(E,F)** Relationships between jumping and safety-seeking behavior during the training and recall sessions (Error bands represent the 95% confidence interval. *N* = 16 per group; CTL, no-stress control; SIS, social isolation stress; M, males; F, females).

Supplementary Figure 3 Linear regressions comparing safety-seeking behavior and the passive defensive mechanisms. **(A,B)** Relationships between freezing and safety-seeking behavior during the training and recall sessions. **(C,D)** Relationships between crouching and safety-seeking behavior during the training and recall sessions. **(E,F)** Relationship between body stretching and safety seeking behavior during the training and recall sessions (Error bands represent the 95% confidence interval. *N* = 16 per group; CTL, no-stress control; SIS, social isolation stress; M, males; F, females).

Supplementary Figure 4 Linear regressions comparing safety-seeking behavior and the other measurements of interest. **(A,B)** Relationship between risk-taking and safety-seeking behavior. **(C,D)** Relationship between grooming and safety-seeking behavior. **(E,F)** Relationship between locomotion and safety-seeking during training and recall (Error bands represent the 95% confidence interval. *N* = 16 per group; CTL, no-stress control; SIS, social isolation stress; M, males; F, females).
