# Supplementary figures and images for: Impacts of social isolation stress in safety learning and the structure of defensive behavior during a spatial-based learning task involving thermal threat
Source: Front Behav Neurosci. 2024 Dec 10;18:1503097. doi: 10.3389/fnbeh.2024.1503097 (PMC11666493; doi:10.3389/fnbeh.2024.1503097)

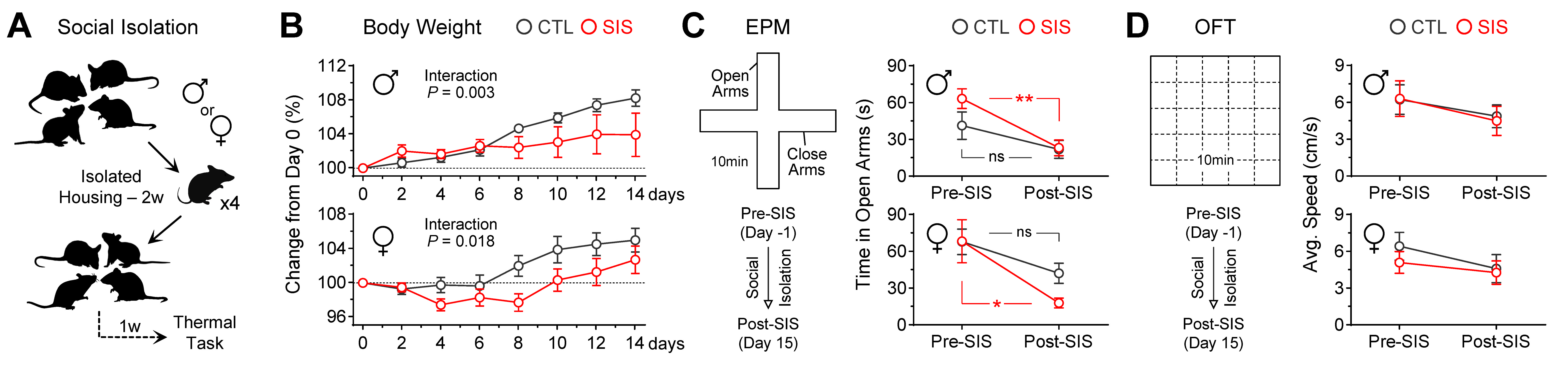

Supplement: Supplementary Figure 1 — Validation of the social isolation procedure. (A) Depiction of timeline of the social isolation stress procedure. (B) Bodyweight measurements every other day during the social isolation procedure. Delays in bodyweight gain represent effectiveness of treatments as emotional stressors. (C) Behavioral testing in the elevated-plus maze (EPM) before and after the social isolation procedure. Significant decays in the time of open arm exploration represent effectiveness of treatments as emotional stressor. While the no-stress control groups showed no significant changes between the pre and post sessions (Males, P = 0.21; Females, P = 0.23), the stress groups exhibited significant decays in open arm exploration (Males, **P = 0.0054; Females, *P = 0.011). (D) Traditional open-field test (OFT) before and after the social isolation procedure. No significant changes were detected for the average speed of motion in the open field (all P’s > 0.53) (N = 8 per group CTL, control; SIS, stress). [file Image_1.tif]

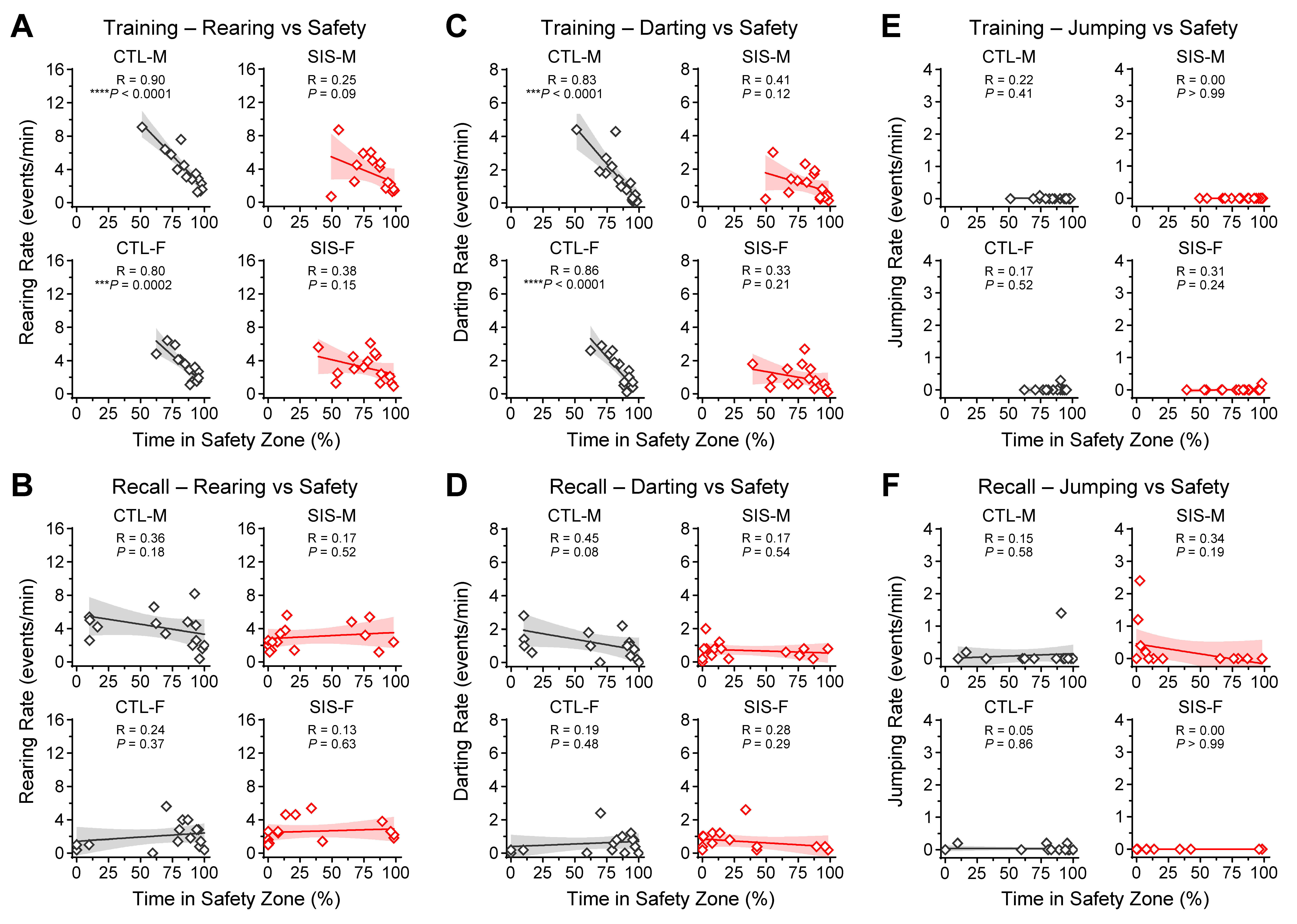

Supplement: Supplementary Figure 2 — Linear regressions comparing safety-seeking behavior and the active defensive mechanisms. (A,B) Relationships between rearing and safety-seeking behavior during the training and recall sessions. (C,D) Relationships between darting and safety-seeking behavior during the training and recall sessions. (E,F) Relationships between jumping and safety-seeking behavior during the training and recall sessions (Error bands represent the 95% confidence interval. N = 16 per group; CTL, no-stress control; SIS, social isolation stress; M, males; F, females). [file Image_2.tif]

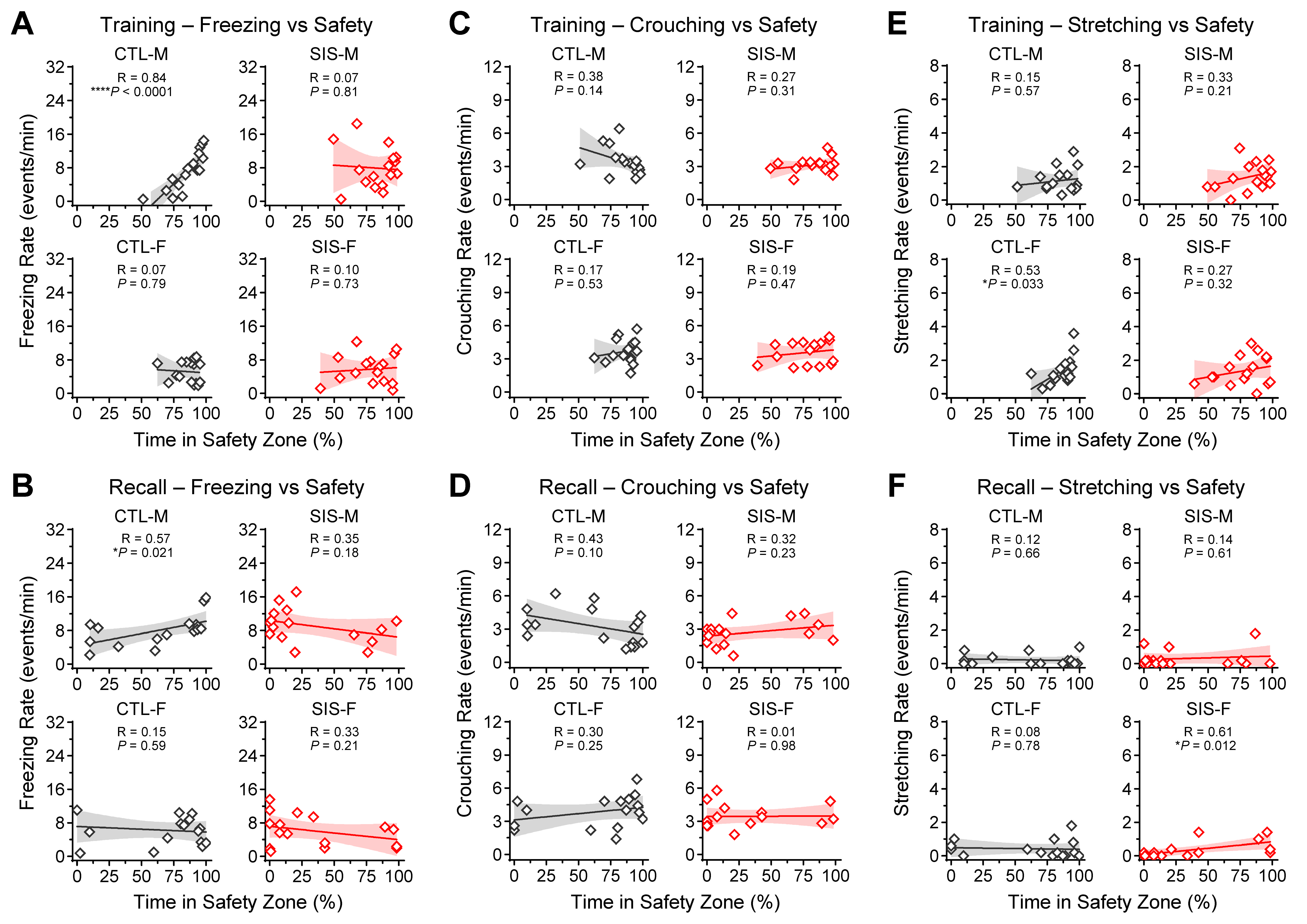

Supplement: Supplementary Figure 3 — Linear regressions comparing safety-seeking behavior and the passive defensive mechanisms. (A,B) Relationships between freezing and safety-seeking behavior during the training and recall sessions. (C,D) Relationships between crouching and safety-seeking behavior during the training and recall sessions. (E,F) Relationship between body stretching and safety seeking behavior during the training and recall sessions (Error bands represent the 95% confidence interval. N = 16 per group; CTL, no-stress control; SIS, social isolation stress; M, males; F, females). [file Image_3.tif]

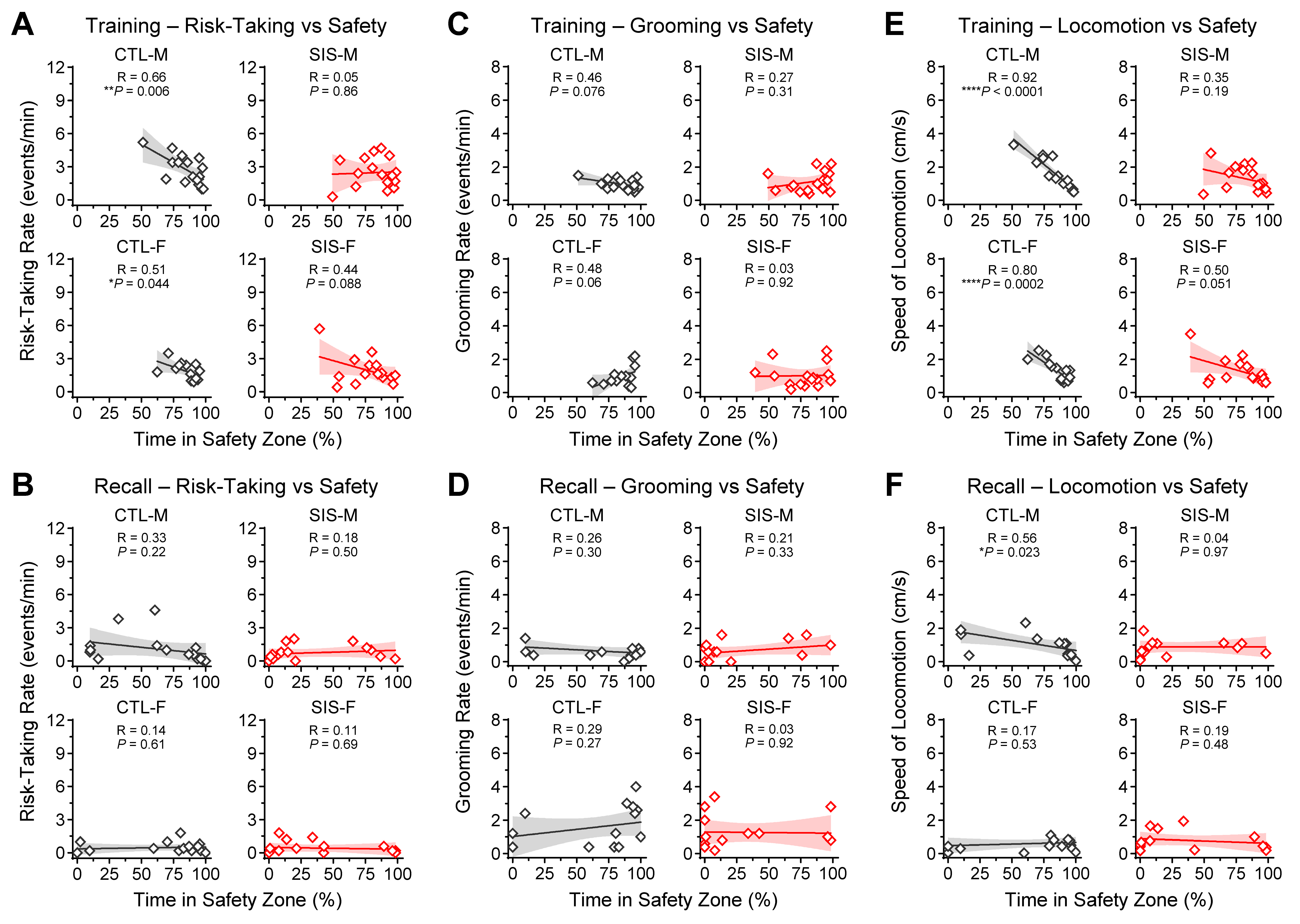

Supplement: Supplementary Figure 4 — Linear regressions comparing safety-seeking behavior and the other measurements of interest. (A,B) Relationship between risk-taking and safety-seeking behavior. (C,D) Relationship between grooming and safety-seeking behavior. (E,F) Relationship between locomotion and safety-seeking during training and recall (Error bands represent the 95% confidence interval. N = 16 per group; CTL, no-stress control; SIS, social isolation stress; M, males; F, females). [file Image_4.tif]
